# A case-control study of risk factors for wound infection in a colorectal unit

**DOI:** 10.1308/003588414X13824511650137

**Published:** 2014-01

**Authors:** K Power, MM Davies, R Hargest, S Phillips, J Torkington, C Morris

**Affiliations:** Department of Colorectal Surgery, Cardiff and Vale University Health Board,UK

**Keywords:** Colorectal, Infection, Risk factors, Case-control study, Obesity

## Abstract

**INTRODUCTION:**

Postoperative wound infections have been responsible for increasing morbidity and are associated with an increased use of hospital resources. Previous studies have identified several risk factors. However, most studies are outdated, and few relate to the era of enhanced recovery and laparoscopic surgery. This study investigated the association between patient and operative factors and the development of postoperative wound infections in colorectal surgery.

**METHODS:**

Patients with documented wound infections or dehiscences were identified from a database of elective and emergency colorectal surgery. Patients with wound infections were matched by operation type to a control group of colorectal patients. Differences in patient and operative factors between case and control group were analysed using conditional logistic regression.

**RESULTS:**

A total of 56 patients with wound infection were identified from 647 operations (8.6%). Fifty-seven per cent were emergency operations and eighty-eight per cent were performed as open surgery or as laparoscopic surgery converted to open. Forty per cent of patients had high ASA (American Society of Anesthesiologists) grades (3 or 4). Multivariate logistical regression showed that obese patients and those having open surgery had the highest risk of infections. The median postoperative hospital stay for patients with wound infections was twice as long as for those patients without wound infections.

**CONCLUSIONS:**

Open surgery and obesity are independent risk factor for wound infections. An increase in laparoscopically performed operations and new strategies for managing wounds in obese patients may help to reduce the rate of wound infection.

Postoperative wound infections cause considerable morbidity and mortality globally. Infection rates in 2011–2012 ranged from 10% in colonic surgery to under 1% in knee prosthesis surgery.^1^ The trends for colonic surgery indicated an upward incidence in the rate of surgical site infections (SSIs) since 2009–2010 (*p*=0.002). The observed trend for small bowel surgery illustrates that inpatient and readmission SSI incidence have been increasing but this did not reach statistical significance (*p*=0.088).^2^ Postoperative wound infections affect the health system financially as each patient with an SSI requires an extra 6.5 days in hospital, increasing healthcare costs to between £814 and £6,626, depending on the type of surgery and the severity of the infection.^3–5^ In the era of enhanced recovery, wound infections are an important complication impeding patient discharge and expending valuable resources.

There is literature that describes the risk factors for postoperative wound infection as: age >65 years, gender, obesity (body mass index [BMI] ≥25kg/m^2^), ASA (American Society of Anesthesiologists) grade ≥3, open surgery,^6^ diabetes mellitus,^7^ wounds classified as dirty/contaminated and emergency operations.^8^ Most of these studies are from 20 or more years ago, and little evidence relates to the era of laparoscopic surgery and enhanced recovery. There is a need for a more current study of risk factors for wound infection. By using a case-controlled study, the relationship between patient factors and postoperative wound infections was investigated in those undergoing elective and emergency operations in a colorectal unit.

## Methods

Patients undergoing elective or emergency colorectal surgery (including small bowel surgery and appendicectomy) between 1 January and 31 December 2011 were identified retrospectively from a prospectively maintained database. This database is updated weekly with the input of medical and nursing staff. All elective patients are enrolled in the enhanced recovery programme, which is based on that described by Kehlet.^9^ While not experiencing the preoperative benefits of the enhanced recovery after surgery programme, emergency patients are managed postoperatively in the same way. These patients had been operated on by one of the five colorectal surgeons in the unit.

Those with a documented abdominal or perineal wound infection during their hospital stay or requiring readmission formed the case group. A wound infection was defined as wound discharge or cellulitis needing antibiotic therapy or intervention (eg wound opening) or spontaneous wound dehiscence. Each patient with a wound infection was matched randomly to two control patients without documented infections who had a comparable operative procedure.

Patient details were recorded. These included surgical diagnosis, co-morbidity and the presence of abdominal obesity. Abdominal obesity was based on the clinical impression of the operating surgeon and a BMI of ≥30kg/m^2^. Operative details collected included type of surgical access, urgency of surgery and level of consultant supervision. Preoperative biochemistry was accessed to determine the presence of anaemia or hypoalbuminaemia. Where positive wound swabs were obtained, the bacteria responsible were recorded.

Differences in patient and operative factors between the case and control groups were analysed using univariate conditional logistic regression to calculate odds ratios with 95% confidence intervals. Those factors that were statistically significantly associated with wound infection were entered into a multivariate analysis to identify independent associations. Linear regression was used to analyse differences in postoperative stay in patients with and without wound infections.

## Results

In 2011, 647 emergency and elective operations were performed with 56 documented wound infections (8.6%). The characteristics of this group of patients are shown in Table 1. The operation with the highest rate of wound infection was abdominoperineal excision (Table 2). Those with wound infections had a significantly longer postoperative hospital stay (13 days) than those without (6 days) (*p*<0.002). Forty patients (71%) with wound infections had wound swabs sent for culture. Of these, 29 (73%) had a positive wound culture. Coliforms were the most frequently cultured organism, being present in 62% of wounds, followed by coryne-form bacteria (13%), coagulase negative *Staphylococcus* (12%) and non-haemolytic *Streptococcus* (11%).

**Table 1:** Study demographics and characteristics of patients with wound infection

Wound infections	56 (8.6%)
Gender	27 female (48%)
Median age	61 years (range: 19–86 years)
ASA grade	
1 and 2	33 (59%)
3 and 4	23 (41%)
BMI ≥30kg/m^2^	14 (25%)
Preoperative anaemia	21 (38%)
Preoperative albumin <30g/l	13 (23%)
Urgency of surgery	32 emergency (43%)
Surgical access	7 laparoscopic (13%)

ASA = American Society of Anesthesiologists;

BMI = body mass index

**Table 2:** Rates of wound infection by type of operation performed in 647 patients

Operation	Total performed	Wound infections
Abdominoperineal resection	14	5 (35.7%)
Hartmann’s procedure	28	6 (21.4%)
Sigmoid colectomy	19	3 (15.7%)
Ileocolic resection	26	4 (15.3%)
Small bowel resection	34	4 (11.7%)
Appendicectomy	81	7 (8.6%)
Subtotal colectomy	29	5 (8.5%)
Other laparotomies (eg adhesiolysis, stoma formation, trauma)	59	4 (6.7%)
Anterior resection	52	3 (5.7%)
Right hemicolectomy	75	4 (5.3%)

On univariate analysis, open surgery, obesity and high ASA grade (3 or 4) had a positive association with wound infection while a cancer diagnosis had a protective association (Table 3). On multivariate analysis, open surgery and obesity remained independent risk factors for wound infection (Table 4).

**Table 3:** Univariate analysis of risk for postoperative wound infection

	Odds ratio	95% CI	*p*-value
Gender	1.0	0.5–1.9	0.9
Old age (>75 years)	0.6	0.3–1.5	0.3
Anaemia	1.0	0.5–2.0	0.9
Low albumin (<30g/l)	1.1	0.5–2.3	0.8
Emergency operations	1.3	0.6–3.0	0.5
Level of supervision	1.1	0.3–4.0	0.8
Open surgery	4.5	1.6–12.2	**0.003**
Stoma formation	1.5	0.6–3.9	0.4
Obesity	4.8	1.7–13.5	**0.003**
High ASA grade (3 or 4)	2.5	1.1–5.5	**0.02**
Previous surgery	1.1	0.5–2.3	0.9
ICU admission	0.9	0.3–2.4	0.86
Cancer	0.4	0.1–1.0	**0.05**

CI = confidence interval;

ASA = American Society of Anesthesiologists;

ICU = intensive care unit

**Table 4:** Multivariate analysis of risk for postoperative wound infection

	Odds ratio	95% CI	p-value
Open surgery	3.83	1.13–11.17	**0.014**
Obesity	3.54	1.16–10.78	**0.026**
High ASA grade	1.54	0.61–3.85	0.356
Cancer	0.35	0.12–1.03	0.057

CI = confidence interval;

ASA = American Society of Anesthesiologists

## Discussion

The group of patients in this study, which included emergency and elective operations, had an overall infection rate of 8.6% compared with the national average of 10% for colorectal surgery.^8^ Open operations and obesity were independent risk factors for postoperative wound infection. Wound infections almost doubled the length of hospital stay and therefore increased healthcare costs. The results demonstrated that coliforms were the most frequently cultured organism, being present in 62% of wounds.

A limitation of this study is that infections identified in the community are not included in departmental figures, leading to an underestimation of the infection risk. However, the most clinically relevant infections are those that cause prolonged hospital stays or readmissions. The Health Protection Agency demonstrated that the percentage of readmissions due to SSIs in 2011 for large bowel and small bowel surgery was 10.3% and 6.8% respectively.^8^

The incidence of postoperative wound infection was significantly higher after open surgery and this finding is supported by other data. A meta-analysis by Abraham *et al* looking at short-term outcomes after laparoscopic colorectal resections demonstrated that the incidence of postoperative wound infection was significantly lower after laparoscopic surgery (3.9%) than after open surgery (8.9%) (*p*<0.005).^10^ Open surgery requires a longer skin incision and more suture material than laparoscopic surgery. This increases the bacterial count in wounds by up to 10^4^ per gram of tissue.^2^ One study reported that the incidence of wound infection after laparoscopic surgery was 2.7% at the trocar site and 10.8% at the site of colorectal removal.^11^

Obesity was a further risk factor for wound infection in this study and may contribute to wound infection through several mechanisms. The primary defence against pathogens is oxidative killing by neutrophils, which is critically dependent on tissue oxygen tension. Incidence of surgical wound infections is therefore directly related to tissue perfusion and oxygenation. Obese patients have decreased tissue oxygen tension at and near the incision site, which increases the risk for SSI.^12^

A surveillance study in 2011 of 197 hospitals in the UK demonstrated that 50% of patients were recorded as having a BMI of ≥25kg/m^2^ for both large and small bowel surgery.^8^ Patients who are morbidly obese present a challenge in achieving adequate antimicrobial concentrations. Evidence suggests these patients should receive a double dose of antibiotics and redosing them is advised if the procedure duration is more than two half-lives of the antibiotic. The use of a higher dose was associated with a decreased rate of SSI in obese patients. Although no definitive recommendation was made, the use of higher doses of prophylactic agents would seem sensible.^13–15^

Topical antibiotics may be beneficial in preventing the incidence of postoperative infections in selective cases such as obese patients undergoing abdominal surgery.^16^ Although it is not advocated in general, there is evidence that it is advantageous for those classified as obese (BMI ≥30kg/m^2^). Further evidence supports that topical kanamycin in the surgical site greatly reduces the incidence of primary infections in the deep subcutaneous space.^17^

Surgical technique, including methods of wound closure can also impact on SSI risk.^18–20^ During the past ten years, numerous clinical studies have supported the use of continuous monofilament fascial closure after laparotomy. Continuous fascial closure reduces major acute wound complications in morbidly obese patients undergoing gastric operations for obesity.^21^ Johnson *et al* prospectively studied the incidence of SSI in 715 patients who underwent a Caesarean section.^22^ The use of subcuticular sutures for skin closure was associated with a significantly higher incidence of SSI than was the use of staples (*p*=0.021).

A similar case-controlled study in Japan involving elective colorectal patients investigated the impact of high pressure irrigation of the patients’ wounds intraoperatively.^23^ The authors found that wound infection occurred in 4% of the patients in the high pressure irrigation group and 18% of the patients in the non-high pressure irrigation group. This difference was significant (*p*=0.041). Furthermore, they stated that for patients with BMIs of ≥25kg/m^2^, they close the wounds with subcuticular sutures after inserting a closed subcutaneous drain to maintain fluid drainage. However, firm evidence supporting the use of a subcutaneous drain after colorectal cancer surgery has yet to be obtained. The insertion of a drain may increase healthcare costs and negatively affect the patient’s ability to walk after surgery.^23^

## Conclusions

Colorectal surgery demonstrates one of the highest rates of SSI. This study found open surgery and obesity were both independent risk factors. Strategies for reducing wound infection should concentrate on methods of wound closure and antibiotic administration in those patients who are morbidly obese. In addition, wound infection rates may be improved by increasing the ability to undertake laparoscopic surgery in obese patients through technological advancements and training.
